# How early environment influences the developing brain and long‐term mental health

**DOI:** 10.1002/jcv2.12230

**Published:** 2024-02-28

**Authors:** Emma Sprooten

**Affiliations:** ^1^ Department of Cognitive Neuroscience Donders Institute for Brain, Cognition and Behaviour Radboud University Medical Center Nijmegen The Netherlands; ^2^ Depertment of Human Genetics Radboud University Medical Center Nijmegen The Netherlands

**Keywords:** brain, environment, mental health, negative life events, neurodevelopment, neuroimaging, resilience, risk, socio‐economic

## Abstract

The March 2024 issue of JCPP Advances features two neuroimaging studies that investigate links between early environmental risk factors for mental health problems, brain development and psychopathology in children and young adults. The papers provide new insights into how adverse environments and negative experiences in childhood increase risk for depression and mental health problems, and how this may or may not be mediated, or moderated, by individual differences in the brain.

## INTRODUCTION

It is common to dichotomise between a biomedical model of mental health research, in which the focus lies on neurobiological, internal causes versus a psychosocial model in which external factors and social interactions are central. However, it is through our brain that we perceive and process our environment. The environment gets under the skin mainly via our brain. Therefore, biomedical and psychosocial mechanisms underlying mental health are highly intertwined. The present issue of JCPP Advances illustrates this, featuring two original research articles that investigate the relationships between environmental factors in childhood, variation in brain anatomy, and mental health problems.

The first paper, by Backhausen et al. (2024), investigated longitudinal links between negative life events in childhood, prefrontal cortex development, and depressive mood in young adulthood. The second article, by Norbom et al. (2024), identified anatomical brain patterns in children that are associated with their parent's income levels and educational attainment, and that may be potential markers of resilience to socio‐economic adversity. These studies complement other recent research (Xu et al., 2023) that looking for links between external risk factors for psychopathology in childhood and individual brain differences, to understand how adverse experiences in childhood can potentially become entrenched to have a long‐lasting impact on mental health throughout life.

## LONG‐TERM EFFECTS OF NEGATIVE LIFE EVENTS IN CHILDHOOD ON BRAIN DEVELOPMENT AND MENTAL HEALTH

Severe childhood adversity and trauma are known risk factors for depression in childhood, as well as in adulthood (Li et al., 2016). Less is known about whether the accumulation of more common, less severe negative experiences also constitute risk factors for depression throughout life. In the longitudinal IMAGEN Consortium cohort (*N* = 321), Backhausen et al. (2024) studied the impact of commonly encountered negative life events in childhood, ranging from illness and death in the family to social and school problems, on depressed mood in young adulthood. Negative life events prior to age 14 were found to cumulatively impact on depressive mood in young adulthood, up to 8 years later. The study thereby provides important new knowledge on the long‐lasting impact of commonly experienced negative life events in childhood, in a general population cohort.

To gain insight into *how* this impression of negative experiences may become engrained and can continue to be impactful over so many years, Backhausen et al. tested whether the experience‐depression association is mediated by altered development of the orbitofrontal cortex (OFC). The OFC is a reasonable candidate to harbour consequences of negative experiences since it plays a role in emotional valuation of external stimuli and memories (Dixon et al., 2017). The OFC has direct anatomical connections with the amygdala and hippocampus, forming a key network that is related to the causes and the remission of depression (Rolls, 2019). Moreover, the OFC and its fibre connections continue maturation into late teens and early twenties. Therefore, this can be a vulnerable period during which interference with OFC development may have long‐lasting effects. In the longitudinal study by Backhausen et al. (2024), OFC thickness was measured four times between age 14 and 22, and showed the expected developmental trajectory of thinning, attributable to synaptic pruning. A thicker OFC at age 14, and a steeper pattern of cortical thinning over time, were associated with more depressed mood at age 22.

Thus, both negative experiences during childhood and adolescent OFC development were associated with depressed mood in adulthood. However, contrary to expectations, the association between negative experiences and depression was *not* mediated by altered development of the OFC. Instead, OFC development and negative life experiences were *independently* associated with depression later in life. The neurobiological mechanisms by which early environment gets under the skin, and remains impactful over many years, appear hard to discern. They may well be subtle and distributed throughout multiple regions and tissues, and heterogeneous depending on the individual and the nature of the experience. Whole‐brain studies that investigate more neuroimaging modalities in larger samples will help to further inform on this fundamental question.

## SEARCHING FOR BRAIN PATTERNS REFLECTING CHILDREN'S RESILIENCE TO SOCIO‐ECONOMIC ADVERSITY

Norbom et al. (2024) report a study of parental education levels and income on general psychopathology in 9758 children aged 9–11. They corroborate previous accounts that socio‐economic adversity is (subtly but significantly) associated with mental health problems in children (Reiss, 2013), and they further investigated the potential role of neuroanatomical variation in individual vulnerability and resilience to socio‐economic adversity.

Previous research already indicated that socio‐economic factors are associated with structural brain measures (Rakesh & Whittle, 2021). However, so far results have been often inconsistent across anatomical locations and neuroimaging measures. Socio‐economic status is a multidimensional construct that reflects a combination of many correlated factors including wealth and education; specific variables like noise levels, toxins, green space; as well as more subjective factors such as perceived social class, social support, and stress. Such a composite measure is unlikely to have very specific effects on circumscribed brain regions or tissues. More likely, the many correlated factors cumulatively and synergistically contribute to many diverse, subtle alterations across brain regions and tissues. What stands out about the present study of Norbom et al. (2024) is that they applied a multivariate method, linked independent component analysis (LICA; LLera Arenas et al., 2018), that is most sensitive to detect such expected non‐specific effects across combinations of regional neuroimaging measures of cortical thickness, surface area, and grey‐white matter contrast.

The takeaway message of their neuroimaging findings is threefold. Firstly, they confirm previous findings that children in more economically affluent families have on average slightly larger cortical surface area, particularly in the prefrontal cortex. Secondly, they found additional associations with grey‐white matter contrast in widespread cortical regions; and with increased thickness, surface area and grey‐white matter contrast in the insula and temporal lobe. Especially the latter finding would be hard to detect in standard unimodal neuroimaging studies as it was driven by the combination of multiple neuroimaging metrics. Thirdly, Norbom et al. found that a pattern of larger prefrontal surface area combined with a smaller occipital surface area attenuated the association of socio‐economic disadvantages with mental health problems, consistent with the possibility that children with a relatively larger prefrontal cortex may be more resilient to socio‐economic disadvantages.

Whilst the idea of a resilience signature in the brain is an intriguingly positive message, the cross‐sectional nature of the study, and the small effect sizes (Cohen's *d* < 0.15), put the findings into perspective. The importance of this study is not so much that it has direct implications for individuals or families, but rather that it makes tangible the subtle, non‐specific but evidently widespread associations of environmental adversity with the brain and mental health.

## IMPLICATIONS AND FUTURE DIRECTIONS

The current issue of JCPP Advances includes two original articles that investigated the associations of environmental risk factors which mental health and brain development. Both studies found associations of early environmental factors and prefrontal brain regions with mental health measures. A strength of the first study by Backhausen et al. (2024) is its longitudinal design spanning across >8 years of adolescence. However, the study did not find evidence for the hypothesised mediating role of OFC development as a mechanism for how negative experiences in childhood cause depression later on. The study was hypothesis‐driven, and it may have missed other potentially mediating brain measures. By contrast, the data‐driven approach by Norbom et al. (2024) indicates that effects of environmental adversity on the brain are not region‐ or modality‐specific. However, this study lacked a prospective component, thereby leaving causal inference to speculation. Future research that combines these multimodal environment‐sensitive brain patterns with the prospective design of Backhausen et al. (2024), would be ideally suited to better understand the longitudinal dynamics, chronicity, and possible causality of environmental influences on brain and mental health throughout development.

Establishing *causal* mechanisms between environmental factors, brain and mental health throughout development, is a major challenge. With the increasing availability of large population studies spurring fully data‐driven research, the web of relevant risk and resilience factors is expanding. Yet, causal inference of risk factors that cluster within families is far from trivial (Cheesman et al., 2023; Plomin, 2022; Sprooten et al., 2022). Since mental health, brain structure, and most environmental factors are heritable, shared genetic vulnerability for psychopathology *and* environmental risks may be partly responsible for their covariation across families. The causal pathways that can explain the observed brain‐environment‐mental health correlations are many. For example,: (a) inherited brain vulnerabilities combined with family‐specific risk environments cause mental health problems; (b) the inheritance of genetic risk factors simultaneously increase the likelihood of encountering environmental risk factors, associated brain patterns, and mental health conditions; (c) mental health problems of the parents cause adverse circumstances for the children which affects their brain development and later mental health problems. Future research that incorporates genetics and neuroimaging to map this gene‐environment interplay within and between families, is promising to map the possible causal effects and their reciprocity throughout development.

The two papers reviewed here are important indicators of what is to come next. The quest to identify which modifiable factors are relevant for whom, in which family, and under which circumstances, requires a deep appreciation and integration of psycho‐social‐societal and biomedical perspectives. By studying the effects of environmental risk factors (i.e. negative life events and socio‐economic adversity) on the developing brain, these two studies contribute to an emerging awareness that studying the brain to understand mental illness is not an isolated endeavour. Neurobiological research can neither afford to ignore external risk factors, nor should it aspire to. Emerging links between brain development, mental health and environment are concrete waypoints towards a better integration of the biomedical, psychosocial, and societal‐political disciplines, that need each other to make an impact, to ultimately benefit mental health in all its variety.

## AUTHOR CONTRIBUTIONS

**Emma Sprooten**: Writing – original draft.

## CONFLICT OF INTEREST STATEMENT

Emma Sprooten is joint editor of JCPP Advances.

## ETHICAL CONSIDERATIONS

None.
